# Mediterranean, DASH, and MIND Dietary Patterns and Cognitive Function: The 2-Year Longitudinal Changes in an Older Spanish Cohort

**DOI:** 10.3389/fnagi.2021.782067

**Published:** 2021-12-13

**Authors:** Stephanie K. Nishi, Nancy Babio, Carlos Gómez-Martínez, Miguel Ángel Martínez-González, Emilio Ros, Dolores Corella, Olga Castañer, J. Alfredo Martínez, Ángel M. Alonso-Gómez, Julia Wärnberg, Jesús Vioque, Dora Romaguera, José López-Miranda, Ramon Estruch, Francisco J. Tinahones, José Lapetra, J. Luís Serra-Majem, Aurora Bueno-Cavanillas, Josep A. Tur, Vicente Martín Sánchez, Xavier Pintó, Miguel Delgado-Rodríguez, Pilar Matía-Martín, Josep Vidal, Clotilde Vázquez, Lidia Daimiel, Cristina Razquin, Oscar Coltell, Nerea Becerra-Tomás, Rafael De La Torre Fornell, Itziar Abete, Carolina Sorto-Sanchez, Francisco Javier Barón-López, Antonio José Signes-Pastor, Jadwiga Konieczna, Antonio Garcia-Rios, Rosa Casas, Ana Maria Gomez-Perez, José Manuel Santos-Lozano, Ana García-Arellano, Patricia Guillem-Saiz, Jiaqi Ni, Maria Trinidad Soria-Florido, M. Ángeles Zulet, Jessica Vaquero-Luna, Estefanía Toledo, Montserrat Fitó, Jordi Salas-Salvadó

**Affiliations:** ^1^Universitat Rovira i Virgili, Departament de Bioquímica i Biotecnologia, and Hospital Universitari San Joan de Reus, Unitat de Nutrició Humana, Reus, Spain; ^2^Institut d’Investigació Sanitária Pere Virgili (IISPV), Reus, Spain; ^3^Consorcio CIBER, M.P. Fisiopatología de la Obesidad y Nutrición (CIBERObn), Instituto de Salud Carlos III (ISCIII), Madrid, Spain; ^4^Toronto 3D (Diet, Digestive Tract and Disease) Knowledge Synthesis and Clinical Trials Unit, Toronto, ON, Canada; ^5^Clinical Nutrition and Risk Factor Modification Centre, St. Michael’s Hospital, Unity Health Toronto, Toronto, ON, Canada; ^6^Nutrition Unit, University Hospital of Sant Joan de Reus, Reus, Spain; ^7^Department of Preventive Medicine and Public Health, University of Navarra, IdiSNA, Pamplona, Spain; ^8^Department of Nutrition, Harvard T.H. Chan School of Public Health, Boston, MA, United States; ^9^Department of Endocrinology and Nutrition, Lipid Clinic, Institut d’Investigacions Biomèdiques August Pi Sunyer (IDIBAPS), Hospital Clínic, Barcelona, Spain; ^10^Department of Preventive Medicine, University of Valencia, Valencia, Spain; ^11^Unit of Cardiovascular Risk and Nutrition, Institut Hospital del Mar de Investigaciones Médicas Municipal d’Investigació Médica (IMIM), Barcelona, Spain; ^12^Department of Nutrition, Food Sciences, and Physiology, Center for Nutrition Research, University of Navarra, IdiSNA, Pamplona, Spain; ^13^Precision Nutrition and Cardiometabolic Health Program, IMDEA Food, CEI UAM + CSIC, Madrid, Spain; ^14^Bioaraba Health Research Institute, Cardiovascular, Respiratory and Metabolic Area, Osakidetza Basque Health Service, Araba University Hospital, University of the Basque Country UPV/EHU, Vitoria-Gasteiz, Spain; ^15^EpiPHAAN Research Group, School of Health Sciences, Instituto de Investigación Biomédica de Málaga (IBIMA), University of Málaga, Málaga, Spain; ^16^CIBER de Epidemiología y Salud Pública (CIBERESP), Instituto de Salud Carlos III, Madrid, Spain; ^17^Instituto de Investigación Sanitaria y Biomédica de Alicante, Universidad Miguel Hernández (ISABIAL-UMH), Alicante, Spain; ^18^Health Research Institute of the Balearic Islands (IdISBa), Palma de Mallorca, Spain; ^19^Department of Internal Medicine, Maimonides Biomedical Research Institute of Cordoba (IMIBIC), Reina Sofia University Hospital, University of Cordoba, Cordoba, Spain; ^20^Department of Internal Medicine, Institut d’Investigacions Biomédiques August Pi Sunyer (IDIBAPS), Hospital Clinic, University of Barcelona, Barcelona, Spain; ^21^Department of Endocrinology, Virgen de la Victoria Hospital, Instituto de Investigación Biomédica de Málaga (IBIMA), University of Málaga, Málaga, Spain; ^22^Department of Family Medicine, Research Unit, Distrito Sanitario Atención Primaria Sevilla, Seville, Spain; ^23^Research Institute of Biomedical and Health Sciences (IUIBS), University of Las Palmas de Gran Canaria and Centro Hospitalario Universitario Insular Materno Infantil (CHUIMI), Canarian Health Service, Las Palmas de Gran Canaria, Spain; ^24^Department of Preventive Medicine and Public Health, University of Granada, Granada, Spain; ^25^Instituto de Investigación Biosanitaria ibs, GRANADA, Granada, Spain; ^26^Research Group on Community Nutrition and Oxidative Stress, University of Balearic Islands, Palma de Mallorca, Spain; ^27^Institute of Biomedicine (IBIOMED), University of León, León, Spain; ^28^Lipids and Vascular Risk Unit, Internal Medicine, Hospital Universitario de Bellvitge, Hospitalet de Llobregat, Barcelona, Spain; ^29^Division of Preventive Medicine, Faculty of Medicine, University of Jaén, Jaén, Spain; ^30^Department of Endocrinology and Nutrition, Instituto de Investigación Sanitaria Hospital Clínico San Carlos (IdISSC), Madrid, Spain; ^31^CIBER Diabetes y Enfermedades Metabólicas (CIBERDEM), Instituto de Salud Carlos III (ISCIII), Madrid, Spain; ^32^Department of Endocrinology, Institut d’ Investigacions Biomédiques August Pi Sunyer (IDIBAPS), Hospital Clinic, University of Barcelona, Barcelona, Spain; ^33^Department of Endocrinology and Nutrition, Hospital Fundación Jimenez Díaz, Instituto de Investigaciones Biomédicas IISFJD, University Autonoma, Madrid, Spain; ^34^Nutritional Control of the Epigenome Group, Precision Nutrition and Obesity Program, IMDEA Food, CEI UAM + CSIC, Madrid, Spain; ^35^Department of Computer Languages and Systems, Universitat Jaume I, Castellon, Spain; ^36^Integrative Pharmacology and Systems Neurosciences, Instituto Hospital del Mar de Investigaciones Médicas (IMIM), Barcelona, Spain; ^37^Division of Pure and Applied Biochemistry, Lund University, Lund, Sweden

**Keywords:** cognition, dietary pattern, Mediterranean diet (MedDiet), DASH diet, MIND diet

## Abstract

**Background and Aims:** Plant-forward dietary patterns have been associated with cardiometabolic health benefits, which, in turn, have been related to cognitive performance with inconsistent findings. The objective of this study was to examine the relationship between baseline adherence to three *a priori* dietary patterns (Mediterranean, DASH, and MIND diets) with 2-year changes in cognitive performance in older adults with overweight or obesity and high cardiovascular disease risk.

**Methods:** A prospective cohort analysis was conducted within the PREDIMED-Plus trial, involving 6,647 men and women aged 55–75 years with overweight or obesity and metabolic syndrome. Using a validated, semiquantitative 143-item food frequency questionnaire completed at baseline, the dietary pattern adherence scores were calculated. An extensive neuropsychological test battery was administered at baseline and 2-year follow-up. Multivariable-adjusted linear regression models were used to assess associations between 2-year changes in cognitive function *z*-scores across tertiles of baseline adherence to the *a priori* dietary patterns.

**Results:** Adherence to the Mediterranean diet at baseline was associated with 2-year changes in the general cognitive screening Mini-Mental State Examination (MMSE, β: 0.070; 95% CI: 0.014, 0.175, *P-trend* = 0.011), and two executive function-related assessments: the Trail Making Tests Part A (TMT-A, β: −0.054; 95% CI: −0.110, − 0.002, *P-trend* = 0.047) and Part B (TMT-B, β: −0.079; 95% CI: −0.134, −0.024, *P-trend* = 0.004). Adherence to the MIND diet was associated with the backward recall Digit Span Test assessment of working memory (DST-B, β: 0.058; 95% CI: 0.002, 0.114, *P-trend* = 0.045). However, higher adherence to the DASH dietary pattern was not associated with better cognitive function over a period of 2 years.

**Conclusion:** In older Spanish individuals with overweight or obesity and at high cardiovascular disease risk, higher baseline adherence to the Mediterranean dietary pattern may be associated with better cognitive performance than lower adherence over a period of 2 years.

## Introduction

Cognitive decline, associated with aging, is a serious public health concern, given the increasing prevalence of neurodegenerative diseases as people are living longer and the proportion of older persons worldwide continues to rise rapidly (United Nations et al., 2020). Globally, dementia affects an estimated 50 million people, and this prevalence is projected to increase over 130 million by 2050 (World Health Organization [WHO], 2019). Epidemiological studies further suggest a negative interaction of aging and obesity with cognitive dysfunction (Kanaya et al., 2009; Hassing et al., 2010). With the prevalence of overweight and obesity affecting an estimated 30% of the adult population and more, there are additional adverse implications for cognition health (Ng et al., 2014; Balasubramanian et al., 2020). Cognitive decline carries a significant social and economic burden, given cognitive impairment and dementia are strong predictors of functional disability and dependence (Petersen et al., 2001). Cognitive decline is a normal part of the aging process; however, the rate of decline may vary depending on the differences in genetic and lifestyle-related factors (Wu et al., 2020).

The potential of modifiable lifestyle factors is important as there are no effective pharmacological agents identified for the improvement of cognition or delay of the progression of cognitive decline (Petersen et al., 2018). Diet is a key lifestyle risk factor. Individual nutrients and foods have been inconsistently associated with cognitive function, including some vitamins, carotenoids, long-chain *n*-3 polyunsaturated fatty acids (PUFAs), such as seafood, and whole foods rich in polyphenols, such as fruits and vegetables, nuts, olive oil, and coffee (Rutjes et al., 2018; Ammar et al., 2020; Brainard et al., 2020). As food is consumed as part of a dietary pattern, it is important to consider the interactions and associations of whole dietary approaches. Three dietary patterns, in particular, are hypothesized to have a beneficial impact on cognitive function: the Mediterranean diet (MedDiet), the Dietary Approaches to Stop Hypertension (DASH), and the MedDiet-DASH Intervention for Neurodegenerative Delay (MIND). The MedDiet and DASH are currently promoted for their cardiovascular benefits (Arnett et al., 2019) yet may also be advisable to benefit cognition in themselves and because of the association of vascular risk factors with dementia risk (Gottesman et al., 2017).

Epidemiological studies and clinical trials have shown a relationship between adherence to MedDiet and cognitive function (Loughrey et al., 2017; Wu and Sun, 2017), and the World Health Organization (WHO) has included this dietary pattern in their guidelines for risk reduction of cognitive decline and dementia; however, the strength of the recommendation is considered conditional (World Health Organization [WHO], 2019). A hybrid of the MedDiet and DASH diet, the MIND diet, is also being promoted for brain health, albeit it has been less extensively investigated in relation to cognition and other cardiometabolic health outcomes (van den Brink et al., 2019). At any rate, dietary recommendations for preventing cognitive decline are still not widely accepted in guidelines due to conflicting and limited evidence. The MedDiet, DASH, and MIND dietary patterns each represent a modifiable lifestyle practice that could aid cognitive performance, yet further evidence is needed to inform cognitive guideline recommendations, as well as assess whether changes may be observed in a period of 2 years.

The aim of this study was to prospectively examine the relationship between baseline adherence to *a priori* dietary patterns, assessed using the MedDiet, DASH, and MIND dietary patterns scores, with 2-year changes in cognitive performance in a large sample of community-dwelling older adults with overweight or obesity at high cardiovascular disease risk.

## Materials and Methods

### Study Design

The present analyses were conducted within the framework of the PREvención con DIeta MEDiterránea (PREDIMED)-Plus trial, as an observational cohort, assessing the longitudinal (2-year) associations between baseline adherence to prespecified dietary patterns and cognitive performance. The PREDIMED-Plus study is an ongoing 6-year, multicenter, randomized, parallel-group and primary prevention trial conducted in Spain. The aim of the trial is to assess the effect of an intensive weight loss intervention program based on an energy-restricted traditional MedDiet and physical activity promotion and behavioral support, on clinical cardiovascular events, than usual care and dietary counseling intervention only with an energy unrestricted MedDiet (control group). More detailed information about the study protocol can be found at http://predimedplus.com/ and elsewhere (Martínez-González et al., 2019).

### Participants

Participants were recruited between October 2013 and December 2016 in 23 Spanish health centers. Eligible participants were community-dwelling adults (55–75 years) with overweight or obesity (BMI: 27–40 kg/m^2^) who met at least three criteria for metabolic syndrome, namely, without stroke, myocardial infarction, or diagnosis of neurodegenerative disease at baseline, according to the International Diabetes Federation and the American Heart Association (Alberti et al., 2009). Participants who had not completed the baseline dietary questionnaires or had reported energy intakes outside the prespecified limits of ≥800 to ≤4,000 kcal/day for men and ≥500 to ≤3,500 kcal/day for women were excluded from these analyses (Willett, 2012). If a given cognitive function assessment was missing, this test was not included in the analysis for that participant.

### Exposure: Dietary Assessments

Trained dietitians assessed dietary intake *via* face-to-face interviews at baseline using a previously validated semiquantitative 143-item food frequency questionnaire (FFQ) (Fernández-Ballart et al., 2010). For each item, a portion size was established, and nine consumption frequencies were available, ranging from “never or almost never” to “≥6 times/day”. Energy and nutrient intakes were obtained using data from Spanish food composition tables and by multiplying the frequency by the portion size and accounting for the duration of the period assessed (Moreiras et al., 2018).

Dietary pattern adherence scores were computed from responses to the FFQ. In the case of the MedDiet, it was determined based on a Mediterranean Diet Adherence Screener (MEDAS) score, ranging from 0 to 14 points, which has been previously validated (Schröder et al., 2011; García-Conesa et al., 2020). The DASH diet was defined using the score developed by Fung et al. (2008), which ranges from 8 to 40 points. For the MIND diet, the score developed by Morris et al. (2015a; Berendsen et al., 2018), which ranges from 0 to 15 points, was used.

### Outcome: Cognitive Assessments

Participants completed a battery of cognitive tasks at baseline and 2 years of follow-up. This battery of neuropsychological tests included Mini-Mental State Examination (MMSE), a commonly used cognitive screening test (Folstein et al., 1975; Blesa et al., 2001); clock-drawing test (CDT) for evaluating visuospatial and visuo-constructive capacity (del Ser Quijano et al., 2004; Aprahamian et al., 2009; Paganini-Hill and Clark, 2011); semantical and phonological verbal fluency tasks (VFT-a and VFT-p, respectively) for assessing verbal ability and executive function (Benton et al., 1994); Trail Making Tests (TMT) parts A and B for executive function assessment, where part A assesses attention and processing speed and part B further examines cognitive flexibility (Llinàs-Reglà et al., 2017); and forward recall and backward recall Digit Span Tests (DST-f and DST-b, respectively) of the Wechsler Adult Intelligence Scale-III (WAIS-III), where DST-f evaluates attention and short-term memory capacity and DST-b tests working memory (Wechsler, 1997; Rossi et al., 2008). Raw scores for each cognitive assessment were standardized using the mean and standard deviation from the baseline population scores, creating *z*-scores. Global cognitive function (GCF) was determined as a composite score of all eight assessments (Shah et al., 2013; Gómez Martínez et al., 2021), adding or subtracting each individual test value based on whether a higher score indicates higher or lower cognitive performance, respectively, using *z*-scores according to the following equation:


GCF=(zMMSE+zCDT+zVFTa+zVFTb+(-zTMTA)+(-zTMTB)+zDSTf+zDSTb)/8.

### Covariate Assessment

Trained staff collected information about sociodemographic (i.e., age, sex, education level, and civil status) and lifestyle (i.e., physical activity, dietary intake, and smoking habits) factors via interviewer-administered questionnaires. Physical activity was assessed using a Spanish validated version of the Minnesota leisure-time physical activity questionnaire (Elosua et al., 1994, 2000). Total daily energy intake was estimated according to data from the FFQ. Anthropometric variables, such as weight and height, were measured by trained staff using calibrated scales and wall-mounted stadiometers, respectively. Body mass index (BMI) was calculated as weight in kilograms divided by height in meters squared. Personal history related to chronic diseases (e.g., hypertension, hypercholesterolemia, and type 2 diabetes) was self-reported or collected from the medical records of participants. Depressive symptomology was evaluated based on Beck’s Depression Inventory-II (BDI-II) with the threshold for depressive status risk established as a score ≥ 14 (Beck et al., 1996; Sanz et al., 2003). The intervention group (treatment or control) and center size (<250, 250 to <300, 300 to <400, ≥400) of the PREDIMED-Plus study were also considered as covariates.

### Statistical Analyses

All statistical analyses were performed using the latest PREDIMED-Plus study dataset generated on December 22, 2020. Data for dietary adherence scores (exposure variables) are presented as median (range). For the covariates and outcome variables, data are shown as percentages and mean ± standard deviation (SD), for qualitative and quantitative descriptive variables, respectively, and as β [95% confidence interval (CI)] for associations. Participants were classified according to tertiles of dietary pattern adherence, and the lowest tertile was used as the reference category. The chi-squared test and one-way ANOVA were used for qualitative and quantitative variables, respectively, to compare baseline characteristics according to dietary pattern adherence score.

Longitudinal associations between adherences to the *a priori* dietary patterns of participants who completed each of the neuropsychological function tests were analyzed separately using multivariate linear regression. All analyses were conducted with robust estimates of the variance to correct for intracluster correlation. Crude and two adjusted models were assessed. The first model was minimally adjusted using established non-modifiable risk factor-related confounders for cognitive function (age, sex) along with intervention arm, study center size, respective baseline cognitive function score, and corrected for clusters (to account for couples living in the same household being randomized as a single unit). The second model was further adjusted for baseline education level (i.e., primary school, secondary school, or college), civil status (i.e., single, divorced or separated, married, or widower), smoking status (i.e., former smoker, never smoked, or current smoker), BMI (kg/m^2^), hypertension (yes/no), hypercholesterolemia (yes/no), diabetes (yes/no), depressive symptomology (yes/no), baseline physical activity (METs min/day), and total energy intake (kcal/day).

The probability *P* for trend across categories of dietary pattern adherence score was calculated using the median value of each category as a continuous variable, and a two-tailed *P-*value < 0.05 was considered statistically significant. Several sensitivity analyses were performed to test the robustness of the findings and identify significant exposure factors to aid in developing priorities for risk mitigation. First, the removal of participants with a baseline MMSE score ≤ 23 indicated possible mild dementia (Dementia Care Central, 2020). Second, alcohol was added as a potential confounder in the models (despite being a component of the MedDiet and MIND patterns) as excessive alcohol intake is considered a risk factor for cognitive decline and dementia (Livingston et al., 2020). Third, analyses were conducted assessing the impact of individual food components of each dietary pattern using linear regression accounting for multicollinearity, if present. Statistical analyses were performed using Stata (14.0, StataCorp LP, TX, United States).

## Results

**FIGURE 1:**
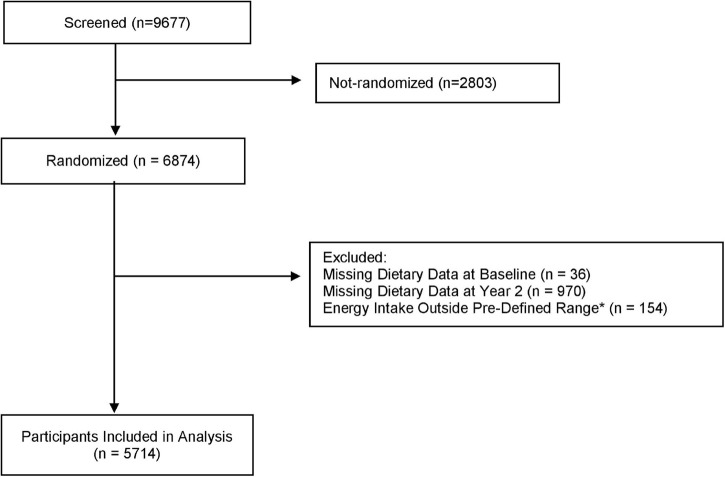
Flow diagram of participants for the analysis of *a priori* dietary pattern adherence and cognitive performance in the PREDIMED-Plus trial. *Energy intakes outside pre-specified limits were identified as ≤800 to ≥4,000 Kcal/d for men and ≤500 to ≥3,500 Kcal/d for women.

Figure 1 provides the flow diagram of participants. This study included a total of 6,647 participants (mean age 65 years, 48% women). Table 1 provides the baseline characteristics of the participants overall and shows the categories representing the lowest and highest adherence to each of the *a priori* dietary pattern scores at baseline. The median (range) of dietary adherence scores for the lowest and highest tertiles of each of the three assessed dietary patterns were 6 (1–7) and 10 (10–14) for the MedDiet (lowest possible score 0, highest possible score 14), respectively; 19 (8–21) and 30 (27–38) for DASH (lowest possible score 8, highest possible score 40), respectively; and 8 (2.5–8.5) and 10.5 (10.0–13.5) for MIND (lowest possible score 0, highest possible score 15), respectively.

**TABLE 1:** Baseline characteristics of the PREDIMED-Plus participants according to categories of highest and lowest baseline adherence (based on tertile categorization^1^) to *a priori* dietary patterns.

**Dietary patterns (score range)**	**Total**	**MedDiet (0–14)**			**DASH (8–40)**			**MIND (0–15)**		
**Trait**		**Lowest**	**Highest**	***P*-value**	**Lowest**	**Highest**	***P*-value**	**Lowest**	**Highest**	***P*-value**
Diet score adherence, Median (Range)		6 (1–7)	10 (10–14)		19 (8–21)	30 (27–38)		8 (2.5–8.5)	10.5 (10.0–13.5)	
Frequency, *n*	6,647	2,415	1,621		2,674	2,141		3,021	1,652	
***Socio-demographic data***										
Age (years)	65.0 ± 4.9	64.5 ± 5.0	65.5 ± 4.8	<0.001	64.1 ± 5.0	65.9 ± 4.6	<0.001	64.7 ± 5.0	65.4 ± 4.9	<0.001
Sex (women)	3,218 (48.4)	1,086 (45.0)	831 (51.3)	<0.001	915 (34.2)	1,372 (64.1)	<0.001	1,374 (45.5)	854 (51.7)	0.001
Education level										
Primary school	3,270 (49.2)	1,201 (49.7)	803 (49.5)		1,213 (45.4)	1,138 (53.2)		1,431 (47.4)	822 (49.8)	
High school	1,918 (28.9)	727 (30.1)	425 (26.2)		849 (31.8)	540 (25.2)		922 (30.5)	447 (27.1)	
College	1,459 (22.0)	487 (20.2)	393 (24.2)	0.010	612 (22.9)	463 (21.6)	<0.001	668 (22.1)	383 (23.2)	0.014
Civil status										
Single, divorced, or separated	858 (12.9)	330 (13.7)	203 (12.5)		323 (12.1)	301 (14.1)		390 (12.9)	238 (14.4)	
Married	5,101 (76.7)	1,834 (75.9)	1,270 (78.4)		2,117 (79.2)	1,577 (73.7)		2,328 (77.1)	1,243 (75.2)	
Widower	688 (10.4)	251 (10.4)	148 (9.1)	0.192	234 (8.8)	263 (12.3)	<0.001	303 (10.0)	171 (10.4)	0.151
***Disease risk factors***										
Body mass index, kg/m^2^	32.5 ± 3.4	32.9 ± 3.5	32.1 ± 3.3	<0.001	32.7 ± 3.4	32.4 ± 3.5	0.012	32.7 ± 3.5	32.4 ± 3.3	0.050
Physical activity (MET min/day)	351.9 ± 329.1	316.3 ± 312.7	408.8 ± 368.1	<0.001	325.7 ± 322.0	382.7 ± 339.9	<0.001	317.2 ± 305.1	396.9 ± 362.8	<0.001
Smoking status										
Never smoked	2,948 (44.4)	1,052 (43.6)	732 (45.2)		965 (36.1)	1,137 (53.1)		1,320 (43.7)	727 (44.0)	
Former smoker	2,876 (43.3)	1,036 (42.9)	715 (44.1)		1,286 (48.1)	801 (37.4)		1,267 (41.9)	753 (45.6)	
Current smoker	823 (12.4)	327 (13.5)	174 (10.7)	0.128	423 (15.8)	203 (9.5)	<0.001	434 (14.4)	172 (10.4)	<0.001
Diabetes	2,047 (30.8)	772 (32.0)	475 (29.3)	0.194	784 (29.3)	659 (30.8)	0.003	949 (31.4)	524 (31.7)	0.140
Hypertension	5,583 (84.0)	2,064 (85.5)	1,339 (82.6)	0.035	2,254 (84.3)	1,775 (82.9)	0.222	2,553 (84.5)	1,379 (83.5)	0.573
Hypercholesterolemia	4,649 (69.9)	1,679 (69.5)	1,116 (68.9)	0.281	1,829 (68.4)	1,513 (70.7)	0.072	2,069 (68.5)	1,198 (72.5)	0.016
Depressive symptoms	1,368 (20.6)	571 (23.6)	304 (18.8)	<0.001	533 (19.9)	477 (22.3)	0.059	683 (22.6)	307 (18.6)	0.001
***Dietary intake***										
Alcohol intake (g/day)	11.0 ± 15.0	11.1 ± 15.2	11.9 ± 16.0	0.004	15.2 ± 17.7	7.0 ± 10.7	<0.001	10.7 ± 15.3	11.6 ± 14.5	0.152
Energy intake (kcal/day)	2,365.2 ± 551.5	2,364.1 ± 597.2	2,434.4 ± 502.3	<0.001	2,467.3 ± 569.1	2,283.5 ± 506.0	<0.001	2,417.6 ± 578.6	2,310.8 ± 512.8	<0.001
***Cognitive function tests***										
GCF^2^	0.02 ± 0.65	0.02 ± 0.66	0.03 ± 0.63	0.852	0.10 ± 0.63	-0.08 ± 0.66	<0.001	0.04 ± 0.64	0.01 ± 0.64	0.543
MMSE	28.22 ± 1.91	28.17 ± 1.94	28.29 ± 1.83	0.154	28.38 ± 1.77	28.01 ± 2.02	<0.001	28.25 ± 1.86	28.18 ± 2.00	0.500
CDT	5.93 ± 1.23	5.91 ± 1.28	5.95 ± 1.20	0.578	6.02 ± 1.19	5.80 ± 1.31	<0.001	5.95 ± 1.22	5.91 ± 1.25	0.566
VFT-a	16.01 ± 4.90	15.88 ± 4.96	16.23 ± 4.94	0.080	16.42 ± 4.95	15.45 ± 4.82	<0.001	16.13 ± 4.92	15.95 ± 4.89	0.205
VFT-p	12.18 ± 4.53	11.96 ± 4.55	12.43 ± 4.57	0.004	12.42 ± 4.50	11.75 ± 4.47	<0.001	12.15 ± 4.56	12.14 ± 4.45	0.572
TMT-A	52.80 ± 28.56	53.01 ± 28.86	52.23 ± 27.33	0.649	49.95 ± 26.31	56.33 ± 31.62	<0.001	52.38 ± 28.22	53.55 ± 29.71	0.411
TMT-B	130.05 ± 72.36	131.96 ± 75.05	127.49 ± 68.05	0.158	122.47 ± 68.08	139.77 ± 72.36	<0.001	128.70 ± 71.89	131.59 ± 72.39	0.365
DST-f	8.79 ± 2.46	8.66 ± 2.44	8.89 ± 2.45	0.013	8.95 ± 2.46	8.56 ± 2.40	<0.001	8.79 ± 2.43	8.73 ± 2.50	0.579
DST-b	5.11 ± 2.22	5.13 ± 2.19	5.09 ± 2.26	0.834	5.33 ± 2.24	4.80 ± 2.15	<0.001	5.18 ± 2.19	4.99 ± 2.26	0.034

*Data are *n* (%) or mean ± SD for categorical and quantitative variables, respectively. The exception is the diet score is represented as mean (range).*

*The chi-squared analysis was used to assess categorical variables and one-way ANOVA for quantitative variables.*

*^1^Highest and lowest dietary pattern adherence categories were determined based on tertiles of baseline data with the highest and lowest adherence groups being tertile 3 and 1, respectively.*

*^2^A composite of *z*-scores was used to calculate GCF using the formula: GCF = (Z_*MMSE*_ + Z_*CDT*_ + Z_*VFT–a*_ + Z_*VFT–b*_ + (–Z_*TMT–A*)_ + (–Z_*TMT–B*_) + Z_*DST–f*_ + Z_*DST–b*_)/8. CDT, clock drawing test; DASH, Dietary Approaches to Stop Hypertension; DST-b, digit span test-backward; DST-f, digit span test -forward; GCF, global cognitive function; MedDiet, Mediterranean dietary pattern; MIND, Mediterranean-DASH Intervention for Neurodegenerative Delay; MMSE, Mini-Mental State Examination; TMT-A, Trail Making Test Part A; TMT-B, Trail Making Test Part B; VFT-a, verbal fluency tasks semantical; VFT-p, verbal fluency tasks phonological.*

A higher percentage of women (*P* ≤ 0.001), older age (*P* < 0.001), higher physical activity (*P* < 0.001), and a tendency toward lower BMI, although not clinically significant (*P* ≤ 0.05), were observed in the highest adherence tertiles for all three dietary patterns. In the DASH and MIND patterns, lower percentages of daily energy intake and current smokers (both *P* < 0.001) were also observed in the highest adherence tertiles of these patterns. Furthermore, higher adherence to the DASH and MIND diets were associated with less alcohol intake (*P* < 0.001) and depression (*P* = 0.001), respectively. For the MedDiet, a lower percentage of participants with depressive symptoms, higher alcohol, and total energy (all *P* < 0.01) was observed in the highest adherence tertile compared with the lowest adherence tertile.

**FIGURE 2:**
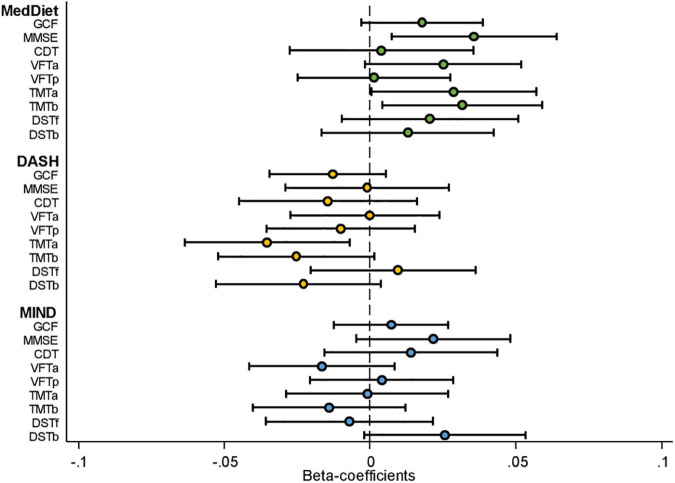
Cognitive function assessment by dietary pattern adherence [standardized beta-coefficients (95% confidence intervals)]. CDT; clock drawing test; DASH; Dietary Approaches to Stop Hypertension; DST-b, digit span test backward; DST-f, digit span test forward; GCF, global cognitive function; MedDiet, Mediterranean dietary pattern; MIND; Mediterranean-DASH Intervention for Neurodegenerative Delay; MMSE, Mini-Mental State Examination; TMT-A, Trail Making Test Part A; TMT-B, Trail Making Test Part B; VFT-a; verbal fluency tasks semantical; VFT-b; verbal fluency tasks phonological. Model presented adjusted for age (in Years), sex, intervention group, centre size (<250, 250 to <300, 300 to <400, ≥400), respective cognitive test score at baseline, baseline education level (primary school, secondary school collage), civil status (single, divorced, or separated, married, and windower), smoking habits (smoker, former smoker, and never smoked), corrected for clusters (to account for couples living in the same household being randomized as a single unit), BMI (kg/m^2^), hypertension (yes/no), baseline physical activity (MET min/week) and total energy intake (kcal/day). For the neurological tests, a positive beta-coefficient value in the figure indicates better cognitive performance according to the associated test.

**FIGURE 3:**
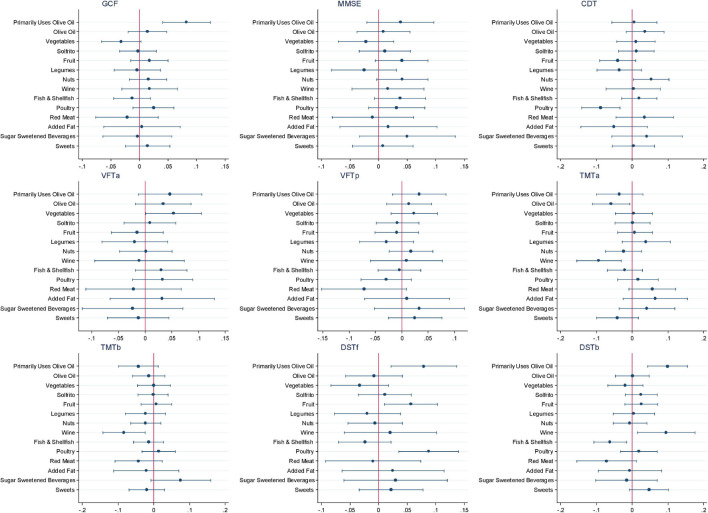
Association of specific dietary components of the Mediterranean dietary pattern (MedDiet) on the cognitive function battery of tests. CDT; clock drawing test; DST-b, digit span test backward; DST-f, digit span test forward; GCF, global cognitive function; MMSE, Mini-Mental State Examination; TMT-A, Trail Making Test Part A; TMT-B, Trail Making Test Part B; VFT-a; verbal fluency tasks semantical; VFT-b; verbal fluency tasks phonological.

Figure 2 and Supplementary Table 1 show the β coefficients (95% CIs) associated with 2-year changes in cognitive assessment *z*-scores across tertiles of *a priori* dietary pattern adherence scores. Results of the fully adjusted linear regression models show a significant association between highest adherence to the MedDiet and 2-year changes in MMSE (β: 0.070; 95% CI: 0.014, 0.175, *P-trend* = 0.011), TMT-A (β: −0.054; 95% CI: −0.110, −0.002, *P-trend* = 0.047), and TMT-B (β: −0.062; 95% CI: −0.116, −0.007, *P-trend* = 0.024). Adherence to the MIND diet was significantly associated with 2-year changes in DST-B (β: 0.058; 95% CI: 0.002, 0.114, *P-trend* = 0.045). No other significant beneficial associations with changes in cognitive performance measured by the different neuropsychological test batteries were observed between adherence to the MedDiet, MIND, or DASH dietary patterns. Conversely, significant associations were observed in the crude models with greater 2-year increases in the DASH diet being associated with lower performance in all nine cognitive tests. Sensitivity analyses, which included assessing only participants with baseline MMSE scores above 23 (as scores 23 and below suggest possible mild dementia or worse), or the addition of alcohol as a potential confounder in the model, did not significantly modify the findings (data not shown). The only modification observed was that including total alcohol intake in the model slightly, but non-significantly, mitigated any negative associations observed between adherence to the DASH diet and changes in cognitive function. Figure 3 shows the impact of all 14 food components of the MedDiet on changes of each individual cognitive test. Of these components, olive oil used as the primary oil was found to be positively associated with changes in global cognitive function, as well as changes in the two DSTs (both forward recall *P* = 0.007 and backward recall *P* ≤ 0.001). Nut intake was significantly and positively associated with an increase in CDT performance (*P* = 0.034) and trended toward beneficially impacting changes in MMSE score (*P* = 0.069). Red wine was also significantly associated with changes in various cognitive function assessments, yet indicating converse findings, where red wine intake may have a beneficial relationship with changes in TMT-A (*P* = 0.004), TMT-B (*P* = 0.006), and DST-b (*P* = 0.020), and a negative association with changes in VFT-a (*P* = 0.048). Likewise, preferably consuming white meat as opposed to red or processed meat showed conflicting findings where a beneficial association was observed with changes in DST-f (*P* = 0.001) and negatively associated with changes in CDT (*P* = 0.001). Fish and shellfish intake was negatively associated with changes in DST-b (*P* = 0.008). Analyses investigating the DASH and MIND diets showed similar associations with nut consumption and wine intake. Additionally, when assessed within the context of the MIND diet, lower consumption of confectionery products was associated with improvements in 2-year changes in GCF (*P* = 0.007), VFT-a (*P* = 0.019), TMT-a (*P* = 0.011), DST-b (*P* = 0.042), and higher red meat intake was associated with worsening changes in TMT-a (*P* = 0.001) and TMT-b (*P* = 0.014) scores.

Table 2 presents the quantity of intake of various dietary components overall and by tertile of dietary pattern adherence score and shows differences between the dietary patterns. When assessing the MedDiet adherence, 94.7% of participants within the highest adherence tertile used olive oil as their primary oil with a mean intake level of 47.4 ± 14.7 g/day, nut intake was 19.9 ± 18.2 g/day, and red wine consumption was on average 59.4 ± 98.1 g/day.

**TABLE 2:** Quantity of dietary intake by tertile of dietary pattern adherence score.

**Dietary pattern**	**Total**	**MedDiet**			**DASH**			**MIND**		
**Baseline intake**		**Lowest adherence**	**Moderate adherence**	**Highest adherence**	**Lowest adherence**	**Moderate adherence**	**Highest adherence**	**Lowest adherence**	**Moderate adherence**	**Highest adherence**
Score, median (range)		6 (1–7)	8 (8–9)	10 (10–14)	19 (8–21)	24 (22–26)	30 (27–38)	8 (2.5–8.5)	9 (9.0–9.5)	10.5 (10.0–13.5)
Olive oil as the primary oil used, *n* (%)	5,266 (79.2)	1,516 (62.8)	2,214 (84.8)	1,536 (94.8)	2,050 (76.7)	1,454 (79.4)	1,762 (82.3)	2,299 (76.1)	1,578 (79.9)	1,389 (84.1)
Olive oil, g/d	39.9 ± 17.0	33.9 ± 16.3	40.7 ± 17.0	47.3 ± 14.7	41.4 ± 16.8	39.3 ± 16.9	38.5 ± 17.2	39.2 ± 17.7	40.1 ± 16.5	40.7 ± 16.4
Vegetables, g/d	328.1 ± 139.9	295.2 ± 132.5	332.1 ± 137.4	370.8 ± 142.5	268.6 ± 111.0	329.1 ± 125.7	401.7 ± 148.4	280.0 ± 123.1	342.9 ± 128.7	398.7 ± 147.7
Green leafy vegetables, g/d	77.8 ± 42.5	68.4 ± 39.3	78.5 ± 42.4	90.5 ± 44.0	64.1 ± 35.4	78.0 ± 40.4	94.6 ± 46.2	63.7 ± 35.4	81.0 ± 39.9	99.7 ± 47.1
Other vegetables, g/d	301.4 ± 125.8	276.3 ± 121.0	303.2 ± 123.4	335.9 ± 128.3	253.8 ± 104.7	303.3 ± 115.5	359.1 ± 133.8	264.4 ± 116.7	314.8 ± 117.5	352.9 ± 130.2
Sofrito, *n* (%)	3,772 (56.8)	985 (40.8)	1,531 (58.6)	1,256 (77.5)	1,528 (57.1)	1,027 (56.1)	1,217 (56.8)	1,707 (56.5)	1,136 (57.6)	929 (56.2)
Fruit, g/d	359.2 ± 206.8	315.2 ± 190.5	365.9 ± 203.8	414.0 ± 220.2	271.5 ± 163.2	365.7 ± 191.6	463.3 ± 218.2	323.7 ± 190.8	367.2 ± 196.1	414.6 ± 232.6
Fruit juice (natural), g/d	59.2 ± 94.5	61.8 ± 101.5	56.6 ± 91.7	59.6 ± 87.6	55.5 ± 92.7	55.6 ± 94.6	66.9 ± 96.1	57.9 ± 94.4	57.9 ± 87.4	63.2 ± 102.3
Berries, g/d	6.0 ± 8.6	5.6 ± 8.8	5.8 ± 8.1	7.0 ± 8.9	4.1 ± 5.7	6.0 ± 8.4	8.4 ± 10.8	4.7 ± 6.7	6.1 ± 7.9	8.3 ± 11.4
Whole grains, g/d	41.3 ± 63.4	33.4 ± 57.1	43.8 ± 64.2	49.0 ± 69.4	19.3 ± 49.2	38.4 ± 60.4	71.2 ± 69.5	22.6 ± 44.3	41.4 ± 62.1	75.3 ± 78.6
Legumes, g/d	20.7 ± 11.2	19.0 ± 10.3	20.8 ± 11.3	23.1 ± 12.1	17.8 ± 9.0	20.3 ± 10.5	24.7 ± 13.1	18.4 ± 9.9	21.3 ± 11.0	24.3 ± 12.7
Nuts, g/d	14.9 ± 17.1	11.1 ± 15.2	15.5 ± 16.8	20.2 ± 18.8	9.8 ± 13.1	14.0 ± 15.8	22.1 ± 20.0	10.5 ± 14.0	15.9 ± 17.3	21.8 ± 19.6
Red Wine, g/d	46.9 ± 90.2	40.4 ± 85.5	44.9 ± 87.2	59.9 ± 100.0	61.5 ± 108.6	43.1 ± 82.0	32.1 ± 65.5	38.3 ± 84.6	49.1 ± 91.7	60.1 ± 96.5
Low-fat dairy, g/d	256.6 ± 201.6	244.0 ± 195.7	258.7 ± 202.1	271.9 ± 208.1	189.3 ± 171.7	267.3 ± 201.1	331.4 ± 208.3	249.2 ± 200.0	256.6 ± 202.0	269.9 ± 203.2
Cheese, g/d	346.2 ± 201.3	348.2 ± 194.3	343.8 ± 202.4	347.0 ± 209.7	300.4 ± 179.4	351.8 ± 203.9	399.5 ± 211.4	355.4 ± 197.1	338.8 ± 203.8	338.0 ± 205.3
Fish and seafood, g/d	102.2 ± 47.7	89.9 ± 46.0	104.1 ± 46.7	117.5 ± 46.8	95.4 ± 46.2	103.0 ± 48.0	110.0 ± 48.1	94.4 ± 47.0	105.6 ± 45.2	112.5 ± 49.5
Poultry, g/d	51.0 ± 28.7	48.3 ± 29.9	51.5 ± 27.2	54.3 ± 28.8	48.0 ± 28.2	51.7 ± 29.0	54.1 ± 28.7	42.3 ± 28.9	54.6 ± 26.6	62.5 ± 25.4
Red meat, g/d	47.4 ± 33.7	55.3 ± 37.8	44.9 ± 31.6	39.8 ± 27.3	60.2 ± 35.9	45.1 ± 30.8	33.4 ± 26.2	52.2 ± 35.2	45.9 ± 32.4	40.5 ± 30.9
Processed meat, g/d	36.4 ± 24.1	40.4 ± 27.7	34.7 ± 22.3	33.3 ± 19.9	44.9 ± 27.4	34.4 ± 21.7	27.5 ± 17.0	39.8 ± 24.2	35.8 ± 23.0	30.9 ± 24.0
Added fats^1^, g/d	2.5 ± 6.4	3.9 ± 9.0	2.0 ± 4.3	1.4 ± 3.4	1.7 ± 3.9	1.5 ± 3.5	1.2 ± 2.9	1.9 ± 4.3	1.2 ± 2.9	0.9 ± 2.3
Sugar-sweetened beverages, ml/d	39.1 ± 89.3	60.6 ± 118.3	29.5 ± 68.9	22.4 ± 55.3	66.3 ± 114.1	30.9 ± 79.2	12.0 ± 37.4	48.9 ± 101.8	33.9 ± 79.2	27.3 ± 72.7
Confectionary/Pastries, g/d	13.7 ± 17.6	18.3 ± 20.9	12.3 ± 16.2	9.0 ± 11.7	15.9 ± 19.5	13.1 ± 16.3	11.5 ± 15.6	18.0 ± 20.8	11.6 ± 14.5	8.5 ± 11.5
Fast/fried foods, g/d	23.8 ± 24.7	28.8 ± 28.4	21.8 ± 22.5	19.5 ± 20.7	30.5 ± 28.4	21.9 ± 22.2	16.9 ± 19.1	31.2 ± 28.6	20.5 ± 20.3	14.1 ± 16.5
Sodium, mg/d	3,281.9 ± 1,012.9	3,350.98 ± 1,064.2	3,237.8 ± 990.4	3,250.3 ± 964.1	3,637.4 ± 1,046.4	3,190.1 ± 957.6	2,916.5 ± 857.5	3,407.1 ± 1,050.5	3,247.2 ± 973.2	3,094.6 ± 956.2

*^1^Added fats include butter, margarine, lard, and cream.*

*Cells filled in gray represent food groupings that are not a main component of the specified dietary pattern.*

*Cells with no fill (i.e., have a white background) represent food groupings that are a main component of the specified dietary pattern.*

*DASH, Dietary Approaches to Stop Hypertension; MedDiet, Mediterranean dietary pattern; MIND, Mediterranean-DASH Intervention for Neurodegenerative delay.*

## Discussion

This study examined the PREDIMED-Plus trial as a longitudinal, observational cohort to evaluate the relationship between adherence to *a priori* dietary patterns and changes in cognitive performance in community-dwelling older adults with overweight or obesity and at high cardiovascular disease risk. Findings suggested that the MedDiet may support cognitive function in older age as significant beneficial associations were observed between greater adherence to the MedDiet with favorable cognitive changes in MMSE, TMT-A, and TMT-B assessments over the follow-up period of 2 years. This represented better general cognitive function, as well as executive function specifically attention and processing speed and cognitive flexibility in those with higher adherence to a MedDiet. Findings also indicated that the MIND diet may be associated with better working memory based on higher adherence being related to higher DST-b assessment. However, the DASH diet was not beneficially associated with 2-year changes in cognitive function in this population with overweight or obesity at high cardiovascular disease risk. The observed advantageous associations between adherence to the MedDiet and cognition align with previous findings presented in systematic reviews and meta-analyses of observational studies suggesting associations between MedDiet adherence with slower cognitive decline, lower risk of dementia (especially Alzheimer’s disease), and reduced conversion of mild cognitive impairment to Alzheimer’s disease (Singh et al., 2014; Loughrey et al., 2017; Wu and Sun, 2017). Recently, a meta-analysis including nine prospective cohort studies reported that high adherence to the MedDiet was associated with a 21% risk reduction in pooled cognitive disorders, in addition to a dose-response with positive findings almost exclusively limited to higher MedDiet adherence (Wu and Sun, 2017). Furthermore, neuroimaging evaluations have found evidence in favor of a protective effect (Karstens et al., 2019). Nevertheless, it is interesting to highlight that previous prospective cohort studies generally had 4 or more years of follow-up, many of which were conducted in relatively healthy, non-Mediterranean populations, with many of the cognitive assessments using screening tests based on criteria to discriminate overall mild cognitive impairment or dementia. Our study also found a positive association between higher adherence to MedDiet and a screening test (MMSE) but also showed specific beneficial associations for executive functioning, including attention and processing speed (TMT-A) and cognitive flexibility (TMT-B). Conversely, a systematic review of randomized controlled trials (nine reports, five unique trials) showed inconsistent findings when comparing a MedDiet with either a waiting list, usual diet, or a low-fat control group for a duration ranging from 10 days to 6.5 years on cognition or brain morphology and function (Radd-Vagenas et al., 2018). However, the authors stated that significant and clinically meaningful effect sizes were found for cognitive composites in the largest and most robust trial, with a duration of 4.1 years, conducted within the context of the PREDIMED trial (Valls-Pedret et al., 2015). Furthermore, in a more recent analysis of a smaller sub-cohort of the PREDIMED-Plus trial evaluating cognition, higher adherence to an energy-reduced MedDiet was associated with greater improvements in memory; however, interpretation of these findings was related to the interplay with weight loss (Soldevila-Domenech et al., 2021). In this study population, we did not find an association for the GCF, which may be explained by the short duration (2 years) and by the broad neuropsychological battery utilized compared with other studies. However, the present findings further support the MedDiet for better cognition while suggesting that beneficial associations may be observable within a shorter timeframe and have applicability for populations at greater risk of cognitive decline (older, with overweight or obesity, and at high risk of cardiovascular disease), which could have implications for improving quality of life. In particular, obesity and its comorbidities are associated with accelerated cognitive decline and impaired cognitive performance including neurodegenerative pathologies, such as dementia, in later life (Dye et al., 2017).

While a significant association was seen in the present analyses with the MedDiet and MIND diet within a shorter follow-up duration with some cognitive function assessments compared with other prospective cohort studies that have been conducted, this relationship was not found with the DASH diet. While MedDiet has been associated with a lower risk of cognitive impairment, it has not always been associated with a slower decline in cognitive function (Keenan et al., 2020). Inconsistent findings have previously been observed with the DASH diet (van den Brink et al., 2019). However, MIND dietary adherence has been associated with better cognitive function across various domains in a systematic review of 13 MIND studies (9 cohorts, 3 cross-sectional, and 1 RCT) evaluating cognitive functioning in older adults (Kheirouri and Alizadeh, 2021). The observed discrepancies with present findings may be related to differences in the types of foods consumed by the study population, and a potential threshold effect related to the amount of each food component consumed, as well as the cognitive tests performed.

The analyzed three dietary patterns each have plant-based foundations, with moderate to high amounts of fish and dairy products, yet they differ in the types and amounts of each dietary component. The MedDiet is typically characterized by high consumption of olive oil, fruits, vegetables, legumes, nuts, cereals, and unsaturated fatty acids; low consumption of meat and saturated fatty acids; low to moderate consumption of dairy products; moderate to high consumption of fish; and a regular, but moderate, intake of wine (Sánchez-Sánchez et al., 2020). The DASH diet shares many similarities yet differs in recommending low-fat dairy and low sodium, besides having fewer specifications (Fung et al., 2008). Based on these two dietary patterns, the MIND diet was developed combining Mediterranean and DASH aspects and incorporating purported neuroprotective foods such as green leafy vegetables and berries (Morris et al., 2015a,b). A potential explanation for our discordant findings may be due to differences in the use of olive oil as the primary oil between tertiles of adherence. In our sample, the use of olive oil was clearly linked with a beneficial association in the GCF composite, as well epidemiological and clinical evidence have suggested improved cognition with olive oil (Millman et al., 2021; Theodore et al., 2021). With any dietary pattern, in addition to observing associations with specific food components, there is also the potential for synergistic effects (Schulz et al., 2021). The present findings suggest such effects given the observed associations for MedDiet with MMSE and TMT-A and TMT-B or the MIND diet with DST-b did not appear to be fully explained by any one component of the dietary pattern; however, further investigation is warranted.

### Potential Mechanisms

The antioxidant, vitamin, probiotic, plant protein, and unsaturated fatty acid content along with low glycemic index/load components of the *a priori* dietary patterns studied have been proposed to possibly affect biological mechanisms of neurocognitive aging (Frisardi et al., 2010). These factors are thought to potentially lead to improved cognition through influencing vascular health and direct promotion of neuroprotection via anti-inflammatory mechanisms and reducing oxidative stress, ameliorating glycemic control, and supporting a favorable microbiome (Caracciolo et al., 2014). Specifically, the observations with the MedDiet and changes in cognitive function may be related to synergistic or individual associations of specific foods, such as olive oil and nuts, due to associations of these foodstuffs with the above-mentioned mechanisms (Viguiliouk et al., 2014; Marcelino et al., 2019; Creedon et al., 2020).

### Limitations and Strengths

There are several limitations to this research. First, the demographic profile of the PREDIMED-Plus cohort, which is composed of predominantly white, older Spanish individuals with metabolic syndrome and overweight or obesity, may limit the generalizability of the results to other populations. However, the homogeneity and the large sample size of the cohort increase the internal validity of the findings by avoiding potential confounding effects of socioeconomic status, educational level, and access to health care. Second, FFQs tend to be limited concerning the variety of foods assessed, as compared to 24-h recalls and food records but are often more likely to reflect usual intake (Willett, 2012). The certainty in the dietary pattern scoring systems utilized may also be limited and experience restrictions due to a potential lack of direct alignment of food items and questions noted in the FFQ with each of the diet score components. FFQs are also prone to misclassification and recall bias as they rely on the memory of participants. This is particularly important in a study of cognition when there could be a decline in cognitive function and memory deficits in the population. However, due to the prospective nature of the study, baseline diet recall is unlikely to have been influenced by cognitive outcomes over the follow-up period, and baseline cognitive function was considered as a confounding variable. Another limitation is that the categorization of dietary pattern adherence was based on sample-specific cutoffs, and there are methodological differences among the various dietary scoring systems available, limiting comparability (van den Brink et al., 2019). Likewise, differences in the types of foods consumed by the study population, and the narrow range in scores between lowest and highest tertiles of intake, may have limited the ability to discern a difference, especially for the MIND pattern. Given that optimal or absolute minimum amounts of key foods for cognitive performance are still unclear, threshold amounts of foods for a neuroprotective effect may not have been reached in the present analyses especially in those consuming lower overall energy intake levels. In terms of cognitive assessments, while the use of a composite domain *z*-score may provide an overall global assessment of cognitive function, the component tests used to create these scores in other studies vary, thus making comparisons difficult. Also, a global screening tool may be less sensitive to detect possible associations due to a potential ceiling effect (Franco-Marina et al., 2010), hence potentially explaining the null associations observed with GCF. Presenting this composite score in addition to the individual cognitive test assessments in this study provides a broader picture of the relationship of these dietary patterns with overall cognition and specific cognitive functions. Additionally, as an observational study, our analysis may be limited by the relatively short duration and is prone to residual confounding from factors not assessed in our models. Specifically, possible genetic interactions, such as with the apolipoprotein E E4 (*ApoE*ε*4*) genotype, which has been associated with cognition, especially in the presence of hypercholesterolemia (Perna et al., 2016), was not able to be accounted for in the current analyses. Finally, a cause-effect relationship could not be determined due to the nature of the study design, as an observational cohort.

Nonetheless, this study is strengthened by the longitudinal analysis conducted in a large cohort using a comprehensive and thoroughly measured battery of cognitive tests that assess various function areas, as well as the use of an FFQ developed and validated for an older Spanish population. The statistical models were also adjusted for multiple sociodemographic, economic, anthropometric, lifestyle, and biological confounders of the association between diet and cognition, while evaluation of the three distinct *a priori* dietary patterns within the same study cohort minimizes the effects of population-specific confounders or effect modifiers.

### Future Directions

Considering the limitations of the present analyses and inconsistencies observed in the literature, studies, especially randomized controlled trials, accounting for relevant genotypes, use of dietary compliance biomarkers such as *via* the development of diet-specific metabolomes, and undertaking standardized neuropsychological assessments including biomarkers and neuroimaging would be useful for future research.

## Conclusion

In older Spanish adults with overweight or obesity, higher adherence to the MedDiet may help mitigate the risk of cognitive decline, specifically as it relates to general and executive cognitive functioning, even over a short (2-year) period.

## Data Availability Statement

Data described in the manuscript, code book, and analytic code will be made available upon request pending application and approval of the PREDIMED-Plus Steering Committee. There are restrictions on the availability of data for the PREDIMED-Plus trial, due to the signed consent agreements around data sharing, which only allow access to external researchers for studies following the project purposes. Requestors wishing to access the PREDIMED-Plus trial data used in this study can make a request to the PREDIMED-Plus trial Steering Committee chair: (JS-S, jordi.salas@urv.cat). The request will then be passed to members of the PREDIMED-Plus Steering Committee for deliberation.

## Ethics Statement

The study was conducted in accordance with the principles of the Declaration of Helsinki. The respective Institutional Review Board (IRB) of all study centres approved the study protocol. The trial was registered at the International Standard Randomized Controlled Trial in 2014 (ISRCTNwww.isrctn.com/ISRCTN8989887089898870). All participants provided written informed consent.

## Author Contributions

MAM-G, DC, JS-S, MF, JAM, AMA-G, JW, JV, DR, JL-M, RE, FJT, JL, JLS-M, AB-C, JAT, VMS, XP, MD-R, PM-M, JV, CV, LD, and ER (all the principal PREDIMED-Plus investigators) contributed to the study concept and design and to data extraction from the participants in the PREDIMED-Plus trial, with SKN, NB, and JS-S, contributing to the study concept and design of the present analyses. SKN, NB, CG-M, NB-T and JS-S performed the statistical analyses. SKN, NB and JS-S drafted the manuscript. All authors reviewed the manuscript for important intellectual content and approved the final version to be published.

## Conflict of Interest

SKN was a volunteer member of the not-for profit group Plant Based Canada. JS-S reported receiving research support from the Instituto de Salud Carlos III, Ministerio de Educación y Ciencia, Departament de Salut Pública de la Generalitat de Catalunya, the European Commission, the United States National Institutes of Health; receiving consulting fees or travel expenses from Danone, California Walnut Commission, Eroski Foundation, Instituto Danone, Nestle, and Abbott Laboratories, receiving non-financial support from Hojiblanca, Patrimonio Comunal Olivarero, the California Walnut Commission, Almond Board of California, La Morella Nuts, Pistachio Growers, and Borges SA; serving on the board of and receiving grant support through his institution from the International Nut and Dried Foundation and the Eroski Foundation; and grants and personal fees from Instituto Danone; Serving in the Board of Danone Institute International. DC reported receiving grants from Instituto de Salud Carlos III. RE reported receiving grants from Instituto de Salud Carlos III, Fundación Dieta Meditarránea and Cerveza y Salud and olive oil for the trial from Fundación Patrimonio Comunal Olivarero and personal fees from Brewers of Europe, Fundación Cerveza y Salud, Interprofesional del Aceite de Oliva, Instituto Cervantes in Albuquerque, Milano and Tokyo, Pernod Ricard, Fundación Dieta Mediterránea (Spain), Wine and Culinary International Forum and Lilly Laboratories; non-financial support from Sociedad Española de Nutrición and Fundación Bosch y Gimpera; and grants from Uriach Laboratories. ER reports grants, personal fees, non-financial support, and others from California Walnut Commission, during the conduct of the study; grants, personal fees, non-financial support and other from Alexion; grants from Amgen and Pfizer; grants, personal fees and other from Sanofi Aventis; personal fees, non-financial support and other from Ferrer International, Danone and Merck Sharp and Dohme, personal fees and other from Amarin, outside the submitted work. The remaining authors declare that the research was conducted in the absence of any commercial or financial relationships that could be construed as a potential conflict of interest.

## Publisher’s Note

All claims expressed in this article are solely those of the authors and do not necessarily represent those of their affiliated organizations, or those of the publisher, the editors and the reviewers. Any product that may be evaluated in this article, or claim that may be made by its manufacturer, is not guaranteed or endorsed by the publisher.
