# Supplementary material for: Mediterranean, DASH, and MIND Dietary Patterns and Cognitive Function: The 2-Year Longitudinal Changes in an Older Spanish Cohort
Source: Front Aging Neurosci. 2021 Dec 13;13:782067. doi: 10.3389/fnagi.2021.782067 (PMC8710807; doi:10.3389/fnagi.2021.782067)
Supplement: Supplementary file 1 [file Data_Sheet_1.PDF]

SUPPLEMENTAL TABLE

Supplemental Table 1. Longitudinal 2-year analysis of multivariable adjusted mean change (β [95% CI]) in cognitive function assessments according to tertiles in level of adherence to each dietary pattern at baseline.

**Dietary Pattern**				**MedDiet**							**DASH**						**MIND**		
**Neurological Assessment[1]**		**Lowest Adherence**		**Modest Adherence**		**Highest Adherence**	**p for trend**	**Lowest Adherence**		**Modest Adherence**		**Highest Adherence**	**p for trend**	**Lowest Adherence**		**Modest Adherence**		**Highest Adherence**	**p for trend**
**Score, median (range)**		**6 (1 to 7)**		**8 (8 to 9)**		**10 (10 to 14)**		**19 (8 to 21)**		**24 (22 to 26)**		**30 (27 to 38)**		**8 (2.5 to 8.5)**		**9 (9.0 to 9.5)**		**10.5 (10.0 to 13.5)**	
**N**		**1904**		**1790**		**914**		**1541**		**1578**		**1489**		**2058**		**1416**		**1134**	
GCF[2]	N	1663		1786		1159		1589	1573		1446			2058	1416		1134		
	Crude model	0 (ref.)	0.010	(-0.057, 0.077)	0.031	(-0.045, 0.107)	0.428	0 (ref.)	-0.159	(-0.228, -0.091)[a]	-0.317	(-0.388, -0.246)[a]	**<0.001**	0 (ref.)	-0.064	(-0.132, 0.005)	-0.027	(-0.099, 0.045)	0.412
	Model 1	0 (ref.)	0.034	(-0.002, 0.070)	0.038	(-0.004, 0.080)	0.059	0 (ref.)	-0.044	(-0.081, -0.006)[a]	-0.022	(-0.062, 0.018)	0.257	0 (ref.)	-0.027	(-0.064, 0.010)	0.022	(-0.018, 0.062)	0.333
	Model 2	0 (ref.)	0.031	(-0.005, 0.067)	0.033	(-0.009, 0.075)	0.095	0 (ref.)	-0.042	(-0.080, -0.004)[a]	-0.027	(-0.067, 0.014)	0.154	0 (ref.)	-0.020	(-0.057, 0.016)	0.023	(-0.017, 0.063)	0.367
MMSE	N	1969		2122		1365		1825	1866		1765			2421	1678		1357		
	Crude model	0 (ref.)	0.046	(-0.014, 0.106)	0.074	(0.008, 0.139)[a]	**0.026**	0 (ref.)	-0.081	(-0.140, -0.021)[a]	-0.178	(-0.241, -0.115)[a]	**<0.001**	0 (ref.)	0.001	(-0.058, 0.061)	0.007	(-0.056, 0.070)	0.822
	Model 1	0 (ref.)	0.059	(0.007, 0.111)[a]	0.077	(0.020, 0.133)[a]	**0.006**	0 (ref.)	0.007	(-0.045, 0.059)	-0.003	(-0.059, 0.053)	0.906	0 (ref.)	0.034	(-0.018, 0.086)	0.036	(-0.017, 0.089)	0.173
	Model 2	0 (ref.)	0.053	(0.002, 0.104)[a]	0.068	(0.017, 0.125)[a]	**0.014**	0 (ref.)	0.013	(-0.039, 0.065)	-0.002	(-0.058, 0.054)	0.941	0 (ref.)	0.044	(-0.007, 0.095)	0.039	(-0.014, 0.092)	0.139
CDT	N	1965		2128		1364		1822	1869.000		1766			2420	1675		1362.000		
	Crude model	0 (ref.)	-0.030	(-0.091, 0.030)	-0.001	(-0.068, 0.066)	0.879	0 (ref.)	-0.098	(-0.160, -0.037)[a]	-0.170	(-0.234, -0.107)[a]	**<0.001**	0 (ref.)	-0.028	(-0.090, 0.034)	0.0003	(-0.064, 0.065)	0.971
	Model 1	0 (ref.)	-0.009	(-0.064, 0.047)	0.021	(-0.042, 0.084)	0.557	0 (ref.)	-0.031	(-0.089, 0.028)	-0.030	(-0.090, 0.031)	0.336	0 (ref.)	-0.008	(-0.066, 0.050)	0.028	(-0.032, 0.087)	0.376
	Model 2	0 (ref.)	-0.015	(-0.071, 0.040)	0.011	(-0.052, 0.074)	0.808	0 (ref.)	-0.026	(-0.085, 0.032)	-0.029	(-0.090, 0.032)	0.351	0 (ref.)	0.002	(-0.056, 0.060)	0.030	(-0.030, 0.090)	0.329
VFT-a	N	2007		2182		1391		1864		1908		1808		2485		1710		1385	
	Crude model	0 (ref.)	0.023	(-0.040, 0.085)	0.072	(0.0005, 0.144)[a]	0.054	0 (ref.)	-0.126	(-0.193, -0.059)[a]	-0.207	(-0.272, -0.141)[a]	**<0.001**	0 (ref.)	-0.044	(-0.108, 0.020)	-0.063	(-0.129, 0.002)	0.055
	Model 1	0 (ref.)	0.042	(-0.006, 0.090)	0.066	(0.012, 0.120)[a]	**0.015**	0 (ref.)	-0.020	(-0.072, 0.032)	0.005	(-0.047, 0.056)	0.870	0 (ref.)	-0.007	(-0.056, 0.042)	-0.032	(-0.083, 0.019)	0.222
	Model 2	0 (ref.)	0.032	(-0.016, 0.079)	0.049	(-0.005, 0.103)	0.069	0 (ref.)	-0.016	(-0.067, 0.035)	-0.004	(-0.055, 0.047)	0.887	0 (ref.)	-0.003	(-0.051, 0.045)	-0.036	(-0.086, 0.014)	0.167
VFT-p	N	2007		2183		1390		1864		1908		1808		2486		1709		1385	
	Crude model	0 (ref.)	0.047	(-0.018, 0.111)	0.055	(-0.018, 0.128)	0.119	0 (ref.)	-0.083	(-0.151, -0.015)[a]	-0.166	(-0.233, -0.098)[a]	**<0.001**	0 (ref.)	-0.032	(-0.098, 0.035)	-0.011	(-0.078, 0.057)	0.726
	Model 1	0 (ref.)	0.028	(-0.020, 0.075)	0.006	(-0.047, 0.058)	0.735	0 (ref.)	-0.028	(-0.078, 0.022)	-0.011	(-0.062, 0.040)	0.664	0 (ref.)	-0.039	(-0.087, 0.009)	0.019	(-0.031, 0.068)	0.526
	Model 2	0 (ref.)	0.020	(-0.026, 0.067)	-0.001	(-0.053, 0.052)	0.928	0 (ref.)	-0.025	(-0.074, 0.025)	-0.02	(-0.071, 0.031)	0.432	0 (ref.)	-0.030	(-0.077, 0.018)	0.015	(-0.035, 0.064)	0.612
TMT-A	N	2004		2176		1390		1862		1908		1800		2485		1705		1380	
	Crude model	0 (ref.)	0.003	(-0.059, 0.064)	-0.060	(-0.129, 0.009)	0.114	0 (ref.)	0.134	(0.076, 0.193)[a]	0.287	(0.221, 0.353)[a]	**<0.001**	0 (ref.)	0.046	(-0.014, 0.105)	0.046	(-0.023, 0.115)	0.177
	Model 1	0 (ref.)	-0.029	(-0.079, 0.021)	-0.079	(-0.135, -0.022)[a]	**0.007**	0 (ref.)	0.036	(-0.012, 0.083)	0.064	(0.008, 0.120)[a]	**0.026**	0 (ref.)	0.020	(-0.028, 0.069)	-0.008	(-0.067, 0.050)	0.810
	Model 2	0 (ref.)	-0.012	(-0.062, 0.037)	-0.060	(-0.117, -0.003)[a]	**0.047**	0 (ref.)	0.034	(-0.013, 0.081)	0.071	(0.014, 0.127)[a]	**0.014**	0 (ref.)	0.023	(-0.031, 0.076)	-0.017	(-0.077, 0.044)	0.979

Continued on page 2.

Supplemental Table 1. Longitudinal 2-year analysis of multivariable adjusted mean change (β [95% CI]) in cognitive function assessments according to tertiles in level of adherence to each dietary pattern at baseline (continued from page 1).

**Dietary Pattern**		**MedDiet**						**DASH**						**MIND**					
**Neurological Assessment**[1]		**Lowest Adherence**	**Modest Adherence**		**Highest Adherence**		**p for trend**	**Lowest Adherence**	**Modest Adherence**		**Highest Adherence**		**p for trend**	**Lowest Adherence**	**Modest Adherence**		**Highest Adherence**		**p for trend**
**Score, median (range)**		**6 (1 to 7)**	**8 (8 to 9)**		**10 (10 to 14)**			**19 (8 to 21)**	**24 (22 to 26)**		**30 (27 to 38)**			**8 (2.5 to 8.5)**	**9 (9.0 to 9.5)**		**10.5 (10.0 to 13.5)**		
**N**		**1904**	**1790**		**914**			**1541**	**1578**		**1489**			**2058**	**1416**		**1134**		
TMT-B	N	1999		2174		1384		1858		1900		1799		2477		1703		1377	
	Crude model	0 (ref.)	-0.039	(-0.102, 0.024)	-0.079	(-0.148, -0.010)[a]	**0.026**	0 (ref.)	0.128	(0.065, 0.190)[a]	0.285	(0.218, 0.352)[a]	**<0.001**	0 (ref.)	0.082	(0.019, 0.145)[a]	0.067	(0.0003, 0.135)[a]	**0.039**
	Model 1	0 (ref.)	-0.054	(-0.104, -0.005)[a]	-0.079	(-0.134, -0.023)[a]	**0.004**	0 (ref.)	0.032	(-0.018, 0.082)	0.045	(-0.009, 0.100)	0.102	0 (ref.)	0.051	(0.002, 0.101)[a]	0.015	(-0.039, 0.069)	0.527
	Model 2	0 (ref.)	-0.037	(-0.086, 0.011)	-0.062	(-0.117, -0.007)[a]	**0.024**	0 (ref.)	0.026	(-0.022, 0.075)	0.051	(-0.003, 0.104)	0.062	0 (ref.)	0.045	(-0.003, 0.094)	0.022	(-0.031, 0.075)	0.382
DST-F	N	1712		1859		1199		1642		1624		1504		2140		1456		1174	
	Crude model	0 (ref.)	0.066	(0.00001, 0.133)	0.072	(-0.004, 0.147)	**0.048**	0 (ref.)	-0.073	(-0.144, -0.002)[a]	-0.155	(-0.224, -0.086)[a]	**<0.001**	0 (ref.)	-0.047	(-0.114, 0.021)	-0.023	(-0.097, 0.050)	0.492
	Model 1	0 (ref.)	0.052	(0.001, 0.104)[a]	0.039	(-0.022, 0.100)	0.159	0 (ref.)	-0.010	(-0.065, 0.046)	0.012	(-0.044, 0.068)	0.683	0 (ref.)	-0.055	(-0.107, -0.003)	-0.010	(-0.069, 0.048)	0.654
	Model 2	0 (ref.)	0.049	(-0.003, 0.100)	0.037	(-0.024, 0.098)	0.184	0 (ref.)	-0.001	(-0.057, 0.054)	0.016	(-0.041, 0.072)	0.584	0 (ref.)	-0.043	(-0.095, 0.009)	-0.007	(-0.065, 0.051)	0.758
DST-B	N	1712		1858		1199		1642		1623		1504		2139		1456		1174	
	Crude model	0 (ref.)	-0.024	(-0.089, 0.042)	-0.006	(-0.082, 0.070)	0.816	0 (ref.)	-0.160	(-0.230, -0.090)[a]	-0.272	(-0.341, -0.202)[a]	**<0.001**	0 (ref.)	-0.034	(-0.101, 0.033)	-0.014	(-0.086, 0.058)	0.673
	Model 1	0 (ref.)	0.013	(-0.038, 0.064)	0.035	(-0.025, 0.095)	0.257	0 (ref.)	-0.061	(-0.116, -0.006)[a]	-0.045	(-0.102, 0.011)	0.111	0 (ref.)	-0.005	(-0.057, 0.047)	0.057	(-0.001, 0.113)	0.062
	Model 2	0 (ref.)	0.005	(-0.046, 0.055)	0.027	(-0.033, 0.087)	0.393	0 (ref.)	-0.057	(-0.111, -0.002)[a]	-0.049	(-0.105, 0.008)	0.089	0 (ref.)	0.006	(-0.045, 0.057)	0.055	(-0.001, 0.112)[a]	0.059

Model 1: Adjusted for age (in years), sex, intervention group, centre size (<250, 250 to <300, 300 to <400, ≥400), corrected for clusters (to account for couples living in the same household being randomized as a single unit), and respective cognitive test score at baseline.
Model 2: Model 1 plus additional adjustment for baseline education level (primary school, secondary school, college), civil status (single, divorced or separated, married, widower), smoking habits (smoker, former smoker, never smoked), BMI (kg/m^2), hypertension (yes/no), hypercholesterolemia (yes/no), diabetes (yes/no), and depressive symptomology (yes/no), baseline physical activity (MET min/week) and total energy intake (kcal/day).
[1]For the neurological tests, a positive value indicates better cognitive performance according to the associated test, except for TMT-A and TMT-B where a negative result indicates better performance.
[2]A composite of z-scores was used to calculate GCF using the formula: $GCF=(Z_{MMSE} + Z_{CDT} + Z_{VFT\text{-}a} + Z_{VFT\text{-}b} + (-Z_{TMT\text{-}A}) + (-Z_{TMT\text{-}B}) + Z_{DST\text{-}f} + Z_{DST\text{-}b}) / 8$.
[a]represents a significant difference (p<0.05) from the reference.
Abbreviations: CDT, Clock Drawing Test; DASH, Dietary Approaches to Stop Hypertension; DST-b, Digit Span test backward; DST-f, Digit Span test forward; GCF, Global Cognitive Function; MedDiet, Mediterranean dietary pattern; MIND, Mediterranean-DASH Intervention for Neurodegenerative delay; MMSE, Mini-Mental State Examination; TMT-A, Trail Making Test Part A; TMT-B, Trail Making Test Part B; VFT-a, Verbal Fluency tasks semantical; VFT-p, Verbal Fluency tasks phonological.
